# Translational potential of human brain charts

**DOI:** 10.1002/ctm2.960

**Published:** 2022-07-20

**Authors:** Saashi A. Bedford, Jakob Seidlitz, Richard A. I. Bethlehem

**Affiliations:** ^1^ Autism Research Centre Department of Psychiatry University of Cambridge Cambridge UK; ^2^ Department of Psychiatry University of Pennsylvania Philadelphia Pennsylvania USA; ^3^ Department of Child and Adolescent Psychiatry and Behavioral Science The Children's Hospital of Philadelphia Philadelphia Pennsylvania USA; ^4^ Lifespan Brain Institute The Children's Hospital of Philadelphia and Penn Medicine Philadelphia Pennsylvania USA; ^5^ Brain Mapping Unit Department of Psychiatry University of Cambridge Cambridge UK

1

Since the advent of magnetic resonance imaging (MRI), researchers have attempted to characterise neuroanatomical alterations and profiles related to myriad human diseases, with the ultimate goal of informing clinical practice. To date, this translational promise has not been fully realised, especially in psychiatric and neurodevelopmental disorders where there are no established pathological indicators.^1^ This may at least partially be attributable to two factors. First, there are large variations in the tools, methods and analyses used. Second, psychiatric conditions are characteristically heterogeneous in presentation, aetiology and biology. Yet, traditional case–control designs assume group homogeneity. An increasingly popular alternative is normative modelling, which explicitly aims to quantify individual variation relative to a normative pattern. Similar to traditional paediatric growth charts, a deviation in, for example, brain morphology can be represented by a centile score, indicating each participant's position within the range of others of the same age and sex.^2^ If the aim is clinical translation, it is vital to first establish a reliable and representative standardised reference. Recently, we aimed to provide such a baseline by mapping the developmental trajectories of multiple commonly studied neuroanatomical phenotypes in a sample of over 100 000 individuals across the full lifespan.^3^

These brain charts present a number of immediate research opportunities to help advance our understanding of a variety of human diseases. First, bringing neuroimaging data into a common space—enabled by harmonisation of large, complex datasets from multiple sources—will allow us to examine group differences at unprecedented scale. This may reveal subtle differences undetectable in smaller datasets, and more broadly fits with the increasing realisation that large sample sizes are pivotal for replicable brain–behaviour relationships.^4^ Second, (per)centile scores derived from these brain charts also provide an immediate opportunity to closely explore inter‐individual variation. For example, we can assess whether more extreme deviations in neuroanatomical scores relate to clinical severity or cognition. Third, centile scoring across neuroanatomical features could enable identification of subgroups that share common patterns of deviation within a clinical classification. For example, in autism there have been findings of both increased and decreased brain volume, which may represent different autistic subtypes. These subgroups may also have significant clinical relevance: having a larger brain has been associated with severe disabilities and poorer prognosis in young autistic boys.^5^ Finally, this approach gives us the ability to explore transdiagnostic neurodevelopmental and ageing patterns. Despite the known phenotypic, genetic and neuroanatomical overlap often observed between conditions, they are typically studied in isolation and often present in different parts of the lifespan. A standard reference space could help delineate both shared and distinct neurobiological signatures across clinical conditions.

Analogous to the use of paediatric growth charts, there is a potential future clinical use case of these brain charts, especially as MRI becomes more affordable and more accessible. Indeed, brain growth charts have already been used in this context.^6^ Centiles provide a common, clinically interpretable language that we hope will in the future aid early diagnoses and thereby improve prognosis or treatment outcomes in psychiatric and neurodegenerative conditions. Neurodevelopmental conditions, where early brain development may deviate from typical trajectories, could become one such application. For example, there is evidence that neuroanatomical alterations in autistic children can be apparent from as early as 6 months, and predict diagnosis at 24 months with high accuracy,^7^ highlighting the potential for brain imaging to inform early identification. Given the high heterogeneity in neurodevelopmental conditions and a lack of understanding of underlying biology or causal mechanisms, providing an exact digital diagnosis is an unrealistic goal. However, normative reference charts may provide an instrument for individualised assessment that could lead to early referral, and fit into a broader clinical evaluation of neurodevelopment. Neurodegenerative conditions, such as Alzheimer's disease or other forms of dementia, where MRI is routinely collected, may be another promising target for clinical translation. Neuroanatomical alterations, such as grey matter atrophy, and divergence of neuroanatomical trajectories associated with dementia are relatively well characterised, and have been shown to be present as early as 10–15 years before symptom onset or diagnosis.^8^ Furthermore, specific neuroanatomical profiles have been linked to variations in Alzheimer's related decline and prognosis.^9^ The ability to track individuals across longitudinal measurements could enable earlier diagnosis and may even predict outcomes. As centile scores are, under normal conditions, stable over time, changes could be indicative of a risk or prodromal phase for neurodegeneration. Changes in centile scores could also be used to monitor progression of the condition over time, and to flag diagnostic transitions, for example, from mild cognitive impairment to dementia.

To conclude, there is still progress to be made before these models can make it into clinical practice. First, progress needs to be made in reconciling neuroanatomical correlates and trajectories of specific conditions, or of subgroups within these conditions. The inherent heterogeneity of these conditions makes this a huge challenge, and may continue to hamper our ability to derive specific predictive clinical information from MRIs despite advances in technology and analysis methods. Another limitation is the lack of accessibility of MRIs, along with challenges of scanning young children or those with neurodevelopmental or psychiatric conditions. Furthermore, despite the large sample size used to derive the current reference, the demographic characteristics mirror known biases in the neuroimaging and general scientific literature, with an overrepresentation of Caucasian, European and North American populations. This limits the generalisability to clinical practice, and future research efforts should endeavour to address this bias, especially in the context of translational research. Finally, there are ethical considerations of consent and data privacy, as well as implications of providing medical advice on currently incurable conditions and the potential for this information to harm rather than help.^10^ This is especially relevant in cases where no known effective cures or treatments exist. Despite these caveats, normative life‐spanning brain charts hold great promise as both a research and clinical tool, and may prove to be an important step towards individualised medicine.
